# An Unusual Case of Tricuspid Valve Infective Endocarditis Caused by Erysipelothrix Rhusiopathiae

**DOI:** 10.7759/cureus.7942

**Published:** 2020-05-03

**Authors:** Pranav Karambelkar, Chaitanya Rojulpote, Austin J Borja, Cathrine Youngs, Abhijit Bhattaru

**Affiliations:** 1 Internal Medicine, The Wright Center for Graduate Medical Education, Scranton, USA; 2 Nuclear Cardiology and Cardiovascular Molecular Imaging, University of Pennsylvania, Philadelphia, USA; 3 Radiology, Perelman School of Medicine at the University of Pennsylvania, Philadelphia, USA; 4 Radiology, Hospital of the University of Pennsylvania, Philadelphia, USA

**Keywords:** infective endocarditis, erysipelothrix rhusiopathiae, zoonosis, tricuspid valve endocarditis

## Abstract

Erysipelothrix rhusiopathiae is an omnipresent commensal in the environment, studied for over a century. It is a zoonotic pathogen known to cause infections in animals and humans. Cases of Erysipelothrix rhusiopathiae in humans have been classified into three distinct entities: localized skin infections, diffuse skin infections, and systemic organ involvement. This particular pathogen is an uncommon cause of endocarditis, with an affinity for the aortic valve. We present a case of Erysipelothrix rhusiopathiae in a patient with involvement of the tricuspid valve.

## Introduction

Erysipelothrix rhusiopathiae is a commonly encountered organism in the environment. Cases in humans involving this organism have been known to cause local and disseminated skin infections, and in rare cases, systemic involvement [1]. Rare cases of Erysipelothrix rhusiopathiae can cause endocarditis. Moreover, this bacteria is known to have a strong affinity for the aortic valve. We present a patient who came to us with overt heart failure. The patient had no typical signs or symptoms suggestive of infective endocarditis but was ultimately found to have significant vegetations on echocardiography. In our report, we describe a rare case of Erysipelothrix rhusiopathiae in a patient with endocarditis and atypical involvement of the tricuspid valve.

## Case presentation

A 47-year-old man with a past medical history of hypertension and alcohol dependence and with a four-week history of progressive bilateral lower extremity edema presented. The patient is a lumberjack and was gradually developing difficulty working long hours. His swelling progressively extended to his knees, prompting a visit to his primary care physician. He underwent a lower extremity duplex in the outpatient setting, which was negative for deep vein thrombosis. The patient was started on oral furosemide but mentioned it did not improve his lower extremity swelling. After undergoing routine lab work with his primary care doctor, the patient was found to have abnormal kidney function and was admitted to the hospital for further evaluation. 

On admission, the patient's vital signs were as follows: temperature of 36.7^o^C, blood pressure of 148/91 mm Hg, heart rate of 72 beats/minute, and respiratory rate of 16/min. Clinically, the patient was volume overloaded with bibasilar lung crackles and 3+ pitting edema in bilateral lower extremities. His laboratory findings were notable for a white count of 26,000 (elevated in part due to patient's oral steroids for unknown reason), anemia, thrombocytopenia, blood urea nitrogen of 56, creatinine of 3.8 (baseline creatinine levels 0.5), hyponatremia, and an N-terminal pro B-type natriuretic peptide (NT-pro-BNP) level of 29,959. Urine studies revealed microscopic hematuria without proteinuria. Electrocardiogram revealed normal sinus rhythm with no acute ST-T wave changes. Further imaging included computed tomography (CT) of the chest, which revealed mild interlobular septal thickening in bilateral lung bases, suggestive of pulmonary edema. CT of the abdomen revealed diffuse gallbladder wall thickening without gallstones with no evidence of ascites. 

At this stage, our preliminary diagnosis was cardio-renal syndrome due to an unknown etiology. The patient was started on intravenous diuretic therapy and oral beta-blockers in light of his acute decompensated heart failure. Additional blood work revealed low complement levels along with elevated cytoplasmic antineutrophil cytoplasmic antibodies (c-ANCA) levels. Antinuclear antibodies, creatine kinase, and anti-streptolysin antibodies were negative. After 48 hours, the patient's blood cultures were positive for gram-positive rods. He was started on empiric treatment with intravenous vancomycin.

**Figure 1:**
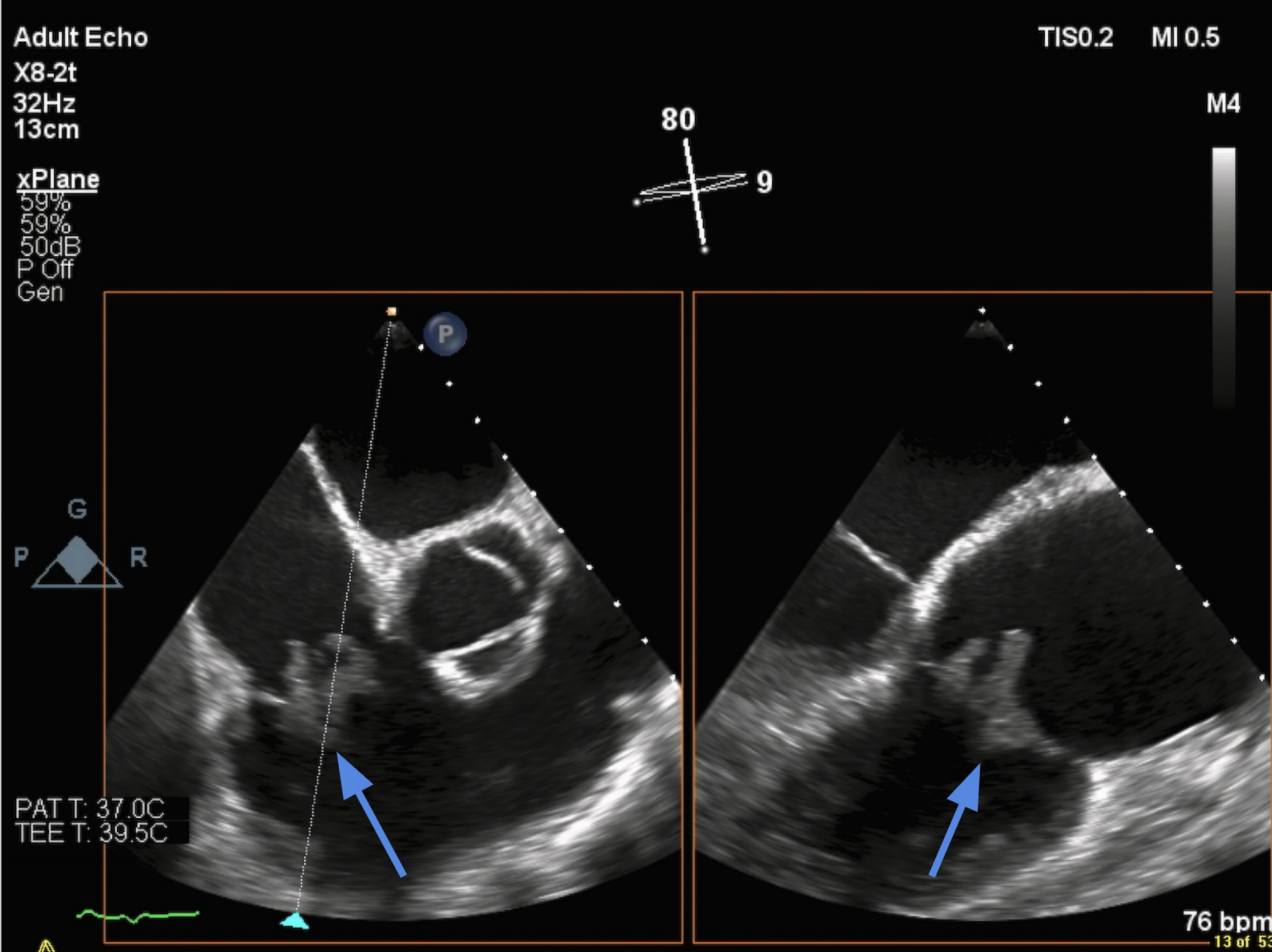
A 2-D echocardiography image of the tricuspid valve showing significant vegetations

Since the patient did not have a baseline heart evaluation on file, a transthoracic echocardiogram was ordered, which revealed a normal ejection fraction with severe tricuspid regurgitation, pulmonary hypertension, and multiple tricuspid valve vegetations (Figure 1).

The most significant vegetation was noted to be 2.6 cm x 1.6 cm in size. At this point, our working diagnosis was acute renal failure, with gram-positive bacteremia secondary to tricuspid valve infective endocarditis. After six days, repeat blood cultures showed gram-positive rods with sensitivities revealing Erysipelothrix rhusiopathiae as the causative agent, resistant to vancomycin and sensitive to Penicillin G and ceftriaxone. The patient's antibiotic regimen was converted to intravenous Penicillin G. Given that he was not a candidate for cardiac surgery, the decision was made to treat him on an outpatient basis with long-term intravenous antibiotics.

## Discussion

Erysipelothrix rhusiopathiae is a gram-positive, rod-shaped, non-sporing bacillus, known to survive for extended periods in the environment. It is considered a zoonotic disease, well-known for its association with occupational exposure. It has been isolated from a wide range of animals, including pigs, sheep, and fish. Reported cases of Erysipelothrix rhusiopathiae have been found frequently in fishers, farmers, butchers, and other occupations involving close exposure to animals [1]. On rare occasions, Erysipelothrix rhusiopathiae has been isolated from domesticated animals, further revealing its ubiquity [2].

Patients who are diagnosed with Erysipelothrix rhusiopathiae manifest with one of three distinct clinical pictures. The most common manifestation is a localized skin infection known as erysipeloid. It presents as local cellulitis, commonly involving the hands [1, 3]. It can usually be differentiated from more common skin infections with its lack of suppuration, lack of edema, and an unusual amount of pain. Diffuse skin infection caused by Erysipelothrix rhusiopathiae is uncommon. Patients usually present with scattered edematous lesions, fever, joint pain, and lymphadenopathy [3]. The third manifestation is systemic bacteremia, although rare, it is known for its high mortality rate. Systemic bacteremia tends to affect the heart, most commonly the aortic valve for unknown reasons [1, 4]. 

Our patient's demographic profile and social history correlated with previous reports of Erysipelothrix rhusiopathiae infections: a middle-aged male with a long-standing history of alcohol use [3, 4]. His occupation as a lumberjack may have increased his risk of contracting this organism. Our patient did admit to animal husbandry in the past, which is a well-known risk factor. However, he did not work in any farm setting over the last ten years. He did, however, admit to hunting and occasionally consuming deer, which may have been the potential source of his infection. The clinical presentation of our patient was unique, given that in the absence of heart disease, he presented with overt volume overload and acute renal failure. He lacked the more classic symptoms of infective endocarditis, including fever, chills, and skin lesions. Our patient did not have any significant past medical conditions increasing his risk for developing infective endocarditis or a history of intravenous drug use. The microscopic hematuria and low complement levels were suggestive of underlying post-infectious glomerulonephritis. However, a transthoracic echocardiogram revealed multiple tricuspid valve vegetations. The most significant vegetation was noted to be 2.6 cm x 1.6 cm in size, suggesting tricuspid valve endocarditis.

In past literature, reported cases of Erysipelothrix rhusiopathiae and endocarditis had shown a proclivity towards the aortic valve [1, 3]. In our case, we felt the most astonishing feature was the isolated involvement of the tricuspid valve. Based on our literature search, there has been a reported case of tricuspid valve involvement in the past [4]. However, to our knowledge, we could not reveal a documented case of Erysipelothrix rhusiopathiae, explicitly involving the tricuspid valve.

The most appropriate method for diagnosing Erysipelothrix rhusiopathiae is by blood culture confirmation. Skin lesions may be biopsied but are not as effective. The major concern when diagnosing Erysipelothrix rhusiopathiae is its similarities to other pathogens, including Listeria monocytogenes, Lactobacillus, and Corynebacterium species. It is crucial to differentiate these species promptly to initiate appropriate antibiotic treatment [1].

The importance of diagnosing Erysipelothrix rhusiopathiae cannot be underestimated. It has a higher mortality rate compared to other pathogens of infective endocarditis [5]. Erysipelothrix rhusiopathiae has also been documented to present as a valvular abscess. Aside from echocardiography, dual imaging modalities such as 18 F-fluorodeoxyglucose positron emission tomography/computed tomography (FDG-PET/CT) has shown to be useful in diagnosing infections [6]. In fact, there has been increasing popularity in utilizing this modality for assessing cardiovascular disorders [7-10]. Nearly all reported cases of Erysipelothrix rhusiopathiae have shown susceptibility to penicillin, cephalosporins, and fluoroquinolones. They are known for their resistance to vancomycin [4]. Our patient did not meet the criteria for the surgical treatment of his valvular lesion and was treated with long term antibiotic therapy [11].

## Conclusions

Erysipelothrix rhusiopathiae is a zoonotic disease, well-known throughout the animal kingdom, yet seldom reported in humans. There has been a great success with advanced farming techniques. However, it is still important to maintain a holistic approach when treating patients with a farming background. This holistic approach to assess patients will help reach a timely diagnosis and initiate appropriate therapy to help reduce mortality. 
